# The Surgical Outcome of Infective Endocarditis in South Africa over 10 Years: A Retrospective Review

**DOI:** 10.3390/jcm13175226

**Published:** 2024-09-03

**Authors:** Riaan Nel, Jacques Janson, Tonya Esterhuizen, Clinton van der Westhuizen

**Affiliations:** 1 Division of Cardiothoracic Surgery, Stellenbosch University and Tygerberg Academic Hospital, Cape Town 7505, South Africa; jjanson@sun.ac.za; 2 Division of Epidemiology and Biostatistics, Stellenbosch University, Cape Town 7505, South Africa; tonyae@sun.ac.za; 3 Division of Medical Microbiology, Stellenbosch University and NHLS Tygerberg Academic Hospital, Cape Town 7505, South Africa; 14519607@sun.ac.za

**Keywords:** infective endocarditis, valve surgery, surgical outcomes, cardiac surgery, valve repair, valve replacement

## Abstract

**Objectives:** There is a paucity of data on the outcome of left-sided cardiac valve surgery for infective endocarditis in South Africa. It is hypothesized that outcomes may be poorer compared to international standards due to differences in disease burden, timing of surgery, organism prevalence, and co-morbidities. **Method:** This is a retrospective study of 160 patients with left heart valve endocarditis who underwent cardiac surgery from January 2010 to December 2019. Demographic, operative, and admission-related parameters were assessed to determine their association with all-cause mortality during the early post-operative (<30 days) and late post-operative (>30 days) periods. **Results:** Early post-operative mortality (<30 days) was 8.8% and late post-operative mortality (>30 days) was 13.1%. Late survival showed 77.5% of the patients were alive with a mean follow-up period of 41 months. Increased age (*p* = 0.04), critical illness (*p* < 0.001), and higher urgency of intervention (*p* < 0.001) were associated with higher early post-operative mortality. Peri-operative organ failure, including cardiac (*p* = 0.025), renal (*p* = 0.016), and respiratory failure (*p* < 0.001), contributed significantly to both early and late mortality. Pre-operative antibiotics for fewer days (*p* = 0.024), ongoing sepsis (*p* = 0.022), and para-valvular extension (*p* = 0.046) were associated with higher early mortality. **Conclusions:** Infective endocarditis is a common indication for cardiac valve surgery in South Africa. Goal-directed medical management and clinical optimization prior to surgery were crucial to achieving better outcomes. Salvage procedures and critical illness with organ failure prior to surgery were associated with poorer outcomes. Despite unique challenges, cardiac surgery for infective endocarditis at Tygerberg Hospital compares favorably to international standards.

## 1. Introduction

### 1.1. Background

Infective Endocarditis (IE) is an infection of the heart valve, endocardial surface, or indwelling intra-cardiac device [1]. The global burden of IE continued to increase from 1990 to 2019, with the incidence in Southern Africa estimated to be approximately 7–9 per 100,000 people. There is substantial heterogeneity in different genders, ages, regions, and organism prevalence making policy development challenging [2,3]. The 1-year mortality rate from IE remains at around 30%, with nearly half of patients with infective endocarditis requiring cardiac valve surgery [1]. Rheumatic Heart disease affects approximately 15 million people on the African continent and is the most common cardiac valve disease at Tygerberg Hospital, with a lower number of patients requiring surgery for infective endocarditis [4]. Cohorts have reported up to 75% of patients with IE have underlying Rheumatic heart valve changes in South Africa [5,6]. In Cape Town, IE contributes significantly to cardiac surgery disease burden and 44.7% of confirmed cases require cardiac valve surgery [5]. Poor socio-economic conditions and poor dental healthcare may be contributing factors [5]. Staphylococci have overtaken Streptococci as the most common cause of the disease [1,5]. Blood culture-negative endocarditis (BCNIE) is a typical finding in all cohorts of IE in South Africa, with recent data suggesting that Bartonella species is a common cause of BCNIE in South Africa [7,8].

### 1.2. Surgical Challenges

Surgical intervention in native valve endocarditis is complex, with surgery often withheld due to medical treatment being considered the best option or the risk of the operation being too high [9]. Surgery for IE is associated with high mortality, but the prognosis of IE surgery seems to be highly variable [10]. The EuroScore II is a valuable tool to identify high-risk IE patients who may not benefit from surgery, but cardiac surgery should only be withheld after thorough consideration [9]. Healthcare in South Africa, including Cardiothoracic Surgery, faces challenges due to resource limitations and patients presenting at an advanced disease stage. Microbial prevalence and patient characteristics create unique challenges in managing patients within the South African setting, in comparison to trends seen in developed countries [11]. Guidelines recommend a multidisciplinary team approach to determine the optimal timing of surgery in patients with IE based on disease severity and patient factors [12,13,14].

Surgical treatment options include valve replacement and repair, with repair showing a better early and late survival advantage in mitral valve endocarditis [15,16]. Mitral valve repairs are often challenging, and various factors have been shown to affect the durability of the repair and subsequent outcomes, including poor ventricular function, the presence of pulmonary hypertension, atrial fibrillation, and the pathophysiology of the mitral incompetence [17].

### 1.3. Factors Influencing Outcomes

Pre-operative predictors of mortality in IE surgery include the EuroScore, age, gender, Left Ventricular Ejection Fraction (LVEF), shock, chronic obstructive pulmonary disease (COPD), creatinine levels, abscess presence, and the isolation of specific organisms. However, cardiac surgery, when indicated, is associated with a good prognosis [9,10,18]. However, the outcome of patients is often associated with the severity of IE, rather than the timing of intervention, and septic shock during initial admission is a notable predictor of poorer outcome [19,20].

HIV’s effect on surgical outcomes associated with infective endocarditis and cardiac valve surgery in South Africa is unclear and needs further research. Surgery for IE has shown excellent results with regard to re-infection and the need for re-operation, with relapse being uncommon [21].

The aim of this research is to review the surgical outcome of patients undergoing left-sided cardiac valve surgery for infective endocarditis at Tygerberg Hospital, from January 2010 to December 2019. The primary outcome is to evaluate the factors influencing mortality at <30 days, >30 days, and the long-term survival of these patients. The secondary outcome is to evaluate pre-operative, peri-operative, and post-operative patient parameters to identify risk factors for morbidity and mortality.

## 2. Patients and Methods

The method used in this study was a retrospective data collection, with a final cohort of 160 patients. Ethical approval was obtained from the Stellenbosch University Health Research Ethics Committee and data were collected from various databases and hospital records. All patients who underwent left-sided cardiac valve surgery between 1 January 2010 and 31 December 2019 were identified using a Cardiothoracic Surgery departmental database search (n = 1158). Inclusion criteria were all confirmed or suspected infective endocarditis patients, undergoing mitral, aortic, or both mitral and aortic valve surgery during this time period. The clinical and operative notes were reviewed, and patients with isolated rheumatic degenerative valve changes were excluded, unless they also had infective endocarditis.

Patients undergoing concomitant surgery were included, but patients undergoing isolated right heart valve surgery were excluded. Prior to 2015, the diagnosis of IE was assessed according to the modified Dukes criteria [22,23]. From 2015 onwards, the Modified Duke’s criteria in conjunction with the updated 2015 European Society of Cardiology (ESC) Infective Endocarditis clinical guidelines were used to confirm eligibility [24]. These guidelines are also endorsed by the European Association for Cardio-Thoracic Surgery (EACTS) as well as the South African Heart Association [25].

Patients with suspected infective endocarditis undergo initial workup and investigation at a peripheral referral hospital. The public sector hospitals in South Africa function on a hierarchical system, with primary clinic care, level 1 local and level 2 regional hospitals. Tygerberg Hospital is a level 3 central academic hospital with a drainage area that comprises about half of the Western Cape province’s population (estimated to be about 7 million people). The majority of level 2 referral hospitals either have their own echocardiogram capabilities or have weekly access to Cardiology consultants, on outreach from Tygerberg Hospital, to aid in diagnosing patients. Diagnosis at peripheral hospitals is usually rapid and will subsequently immediately be assessed by the Cardiologist on Call at Tygerberg regarding the urgency of transfer or further intervention required. If further surgical intervention is likely required, the patient is transferred immediately to Tygerberg Academic Hospital to receive a confirmation of diagnosis, a formal assessment by the Division of Cardiology, and a transthoracic echocardiogram (TTE), according to the ESC guidelines [26]. Should further detail be required, a transoesophageal echocardiogram (TOE), cardiac MRI, or PET CT may also be included in the work-up, but are not considered routine investigations. A multi-disciplinary heart meeting is held weekly to discuss surgical considerations, and a treatment plan is decided upon [13]. This team includes Cardiologists, Cardiothoracic surgeons, Microbiologists, and Infective Disease specialists. Intra-operatively, a final decision regarding valve repair or replacement is made by the consultant Cardiothoracic surgeon, after thorough debridement of infected tissue has been completed, and the mechanism of incompetence evaluated.

Tygerberg Hospital’s Electronic Content Management (ECM) system and physical hospital files were used to capture clinical and Intensive Care Unit (ICU) data. Echocardiography (Echo) data were reviewed using the dedicated Echo-PACS system, and laboratory data were obtained and verified using the National Health Laboratory Service (NHLS) TrackCare and wwDisa pathology interfaces. The outcome of patients was reviewed using several methods, including reviewing clinical data on ECM, reviewing recent blood and INR results on TrackCare, reviewing Echo PACS for follow-up visits and imaging, and reviewing the hospital administration system (Clinicom) for admission dates, discharge dates, and records of mortalities.

Data were imported into IBM SPSS software (version 29) from REDcap^®^. Continuous variables that were normally distributed were summarized using mean and standard deviation, and those that were not normally distributed were expressed as median and interquartile ranges (IQR). Categorical variables were reported as frequency counts and percentages. One-way ANOVA or Kruskal–Wallis tests were used for comparative analysis, and Chi-Square tests or Fisher’s exact 2-sided tests were used to test associations between variables, with a *p*-value of less than 0.05 being considered significant. Kaplan–Meier graphs were used for survival analysis.

Critical pre-operative state and timing of surgery classification were determined by using the EuroScore II scoring system definitions. Patients who require surgery on the current admission for medical reasons and cannot be discharged without a definitive procedure are regarded as urgent procedures. An operation is deemed an emergency if it is carried out before the beginning of the next working day after the decision to operate was made. A salvage procedure is defined as a patient requiring cardiopulmonary resuscitation (external cardiac massage) enroute to the operating theatre or prior to the induction of anesthesia.

See “Supplementary Materials” for further details in relation to the conduction of this study.

## 3. Results

In the patient cohort, the mean age was 37.3 years with a male prevalence of 70% (n = 112). The average EuroScore II was 4.8%, body mass index (BMI) 23.0 kg/m^2^, and body surface area (BSA) 1.75 m^2^. The most common co-morbidities were smoking (46.4%, n = 70), hypertension (27.2%, n = 41), and chronic lung disease (16.6%, n = 25). Acute infective endocarditis was confirmed in 75% (n = 120) of patients at surgery, with isolated mitral and aortic valve interventions required in 46.3% (n = 74) and 37.5% (n = 60) of cases, respectively. In 16.3% (n = 26), both the mitral and aortic valves were involved and addressed. Of all the mitral valves intervened on, 33.0% (n = 33) were repaired and 67% (n = 67) required replacement. For aortic valve interventions, 96.5% (n = 83) were replaced and only 3 cases were deemed suitable for repair techniques.

The median days from admission to surgery was 18.5 days (IQR 6.0–42.0) and the median hospital stay after surgery was 29.0 days (IQR 16.0–43.0), totaling 54.0 days (IQR 37.3–75.0). Most surgeries were first-time valve surgeries (89.4%, n = 143), with native valve endocarditis being the most prevalent (89.3%, n = 143). A total of 56.3% (n = 90) required urgent surgery, and 68.1% (n = 109) had cardiac failure at the time of surgery. A total of 42.1% (n = 67) of patients developed acute kidney injury peri-operatively. Inotropic and respiratory support were required in 25.0% (n = 40) and 17.5% (n = 28), respectively. Important demographic and basic surgical admission parameters and the effect on mortality are set out in Table 1.

After valve surgery, the median intubation time was 18 h (IQR 14–19 h) and the average ICU blood loss within the first 24 h was 350 mL (IQR 200–600 mL). The common early surgical complications included significant blood loss in 32.7% (n = 52) of patients, hospital-acquired pneumonia in 16.4% (n = 26) patients, and cerebrovascular events in 8.2% (n = 13) patients. The follow-up period of 41.0 months (IQR 23.0–70.0 months) showed an infective endocarditis recurrence rate of 6.9% (n = 11) and repeat valve surgery was required in 8.1% (n = 13) of patients. Blood cultures were positive in 47.5% (n = 76) of patients, with Staphylococci being the most prevalent organism identified in 20.6% (n = 33) of patients. All cultures (including both blood and valve cultures) were negative in 41.9% (n = 67) of the patients operated on. Specialized organism-directed Polymerase Chain Reaction (PCR) testing was not routinely carried out on all samples.

In the sub-group analysis in Table 2, a statistically significant difference in mean age was found between survivors and early and late mortality groups (*p* = 0.04). Functional classification according to the New York Heart Association (NYHA) grading showed an association with mortality, with worse clinical status in both the early and late period mortality compared with the survivors (*p* < 0.001). EuroScoreII also showed a statistically significant association with early mortality (*p* < 0.001), but not with late mortality (*p* = 0.306). Comorbidities, gender, and the type of valve IE did not yield different outcomes.

The effects of important pre-operative factors on early and late mortality are tabulated in Table 3. The most notable factors influencing early and late mortality were organ dysfunction and critically ill patients. This can be demonstrated by increased mortality in critically ill patients (*p* < 0.001) and patients undergoing more urgent intervention (*p* < 0.001). Cardiac function and pre-operative laboratory parameters were not found to be statistically significant.

Surgical factors, indications for surgery, and concomitant procedures, as noted in Table 4, showed no significant influence on the mortality in this patient cohort.

**Figure 1:**
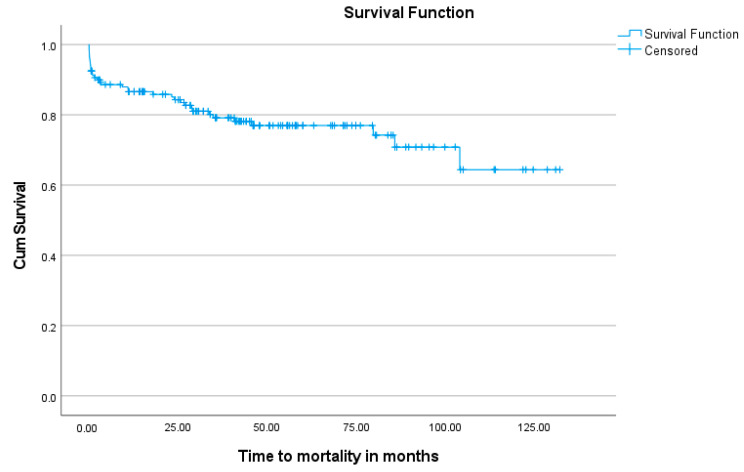
Kaplan–Meier graph indicating the probability of survival at various time points. Mortality or last confirmed alive date are indicated as an event in time. The non-events (alive), but where someone did not make it to the end of this study (dropouts), are censored. The graph shows the proportion of the cohort that is still alive at each point and is calculated with a denominator excluding the censored. By the last death, the probability of survival was 64.4%.

**Figure 2:**
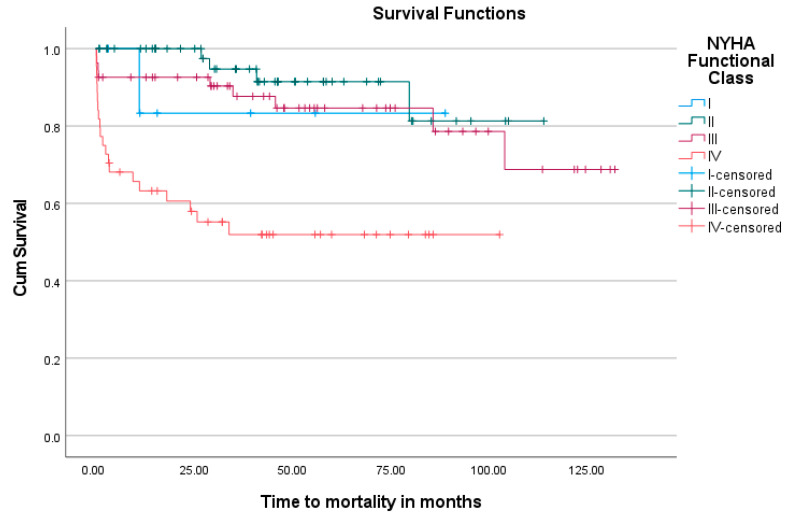
Kaplan–Meier graph indicating the probability of survival at various time points in respect to the different New York Heart Association classifications (NYHA).

**Figure 3:**
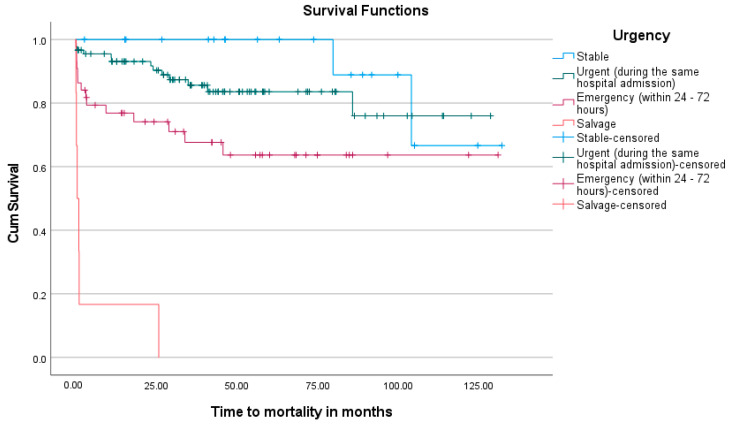
Kaplan–Meier graph indicating the probability of survival at various time points in respect to the Urgency of intervention required, according to the EuroScoreII definitions.

Figure 1 shows a Kaplan–Meier analysis of the overall survival over time in the cohort. The non-events (alive), but where someone did not make it to the end of this study (dropouts) are censored, thus the proportion alive at each point is calculated with a denominator excluding the censored. During the mean follow-up period of 41.0 months, 77.5% of patients were still confirmed to be alive. The effect that the urgency of intervention and clinical status of the patient at surgery has on mortality and late survival can also be visualized in the Kaplan–Meier graphs shown in Figure 2 and Figure 3.

**Figure 4:**
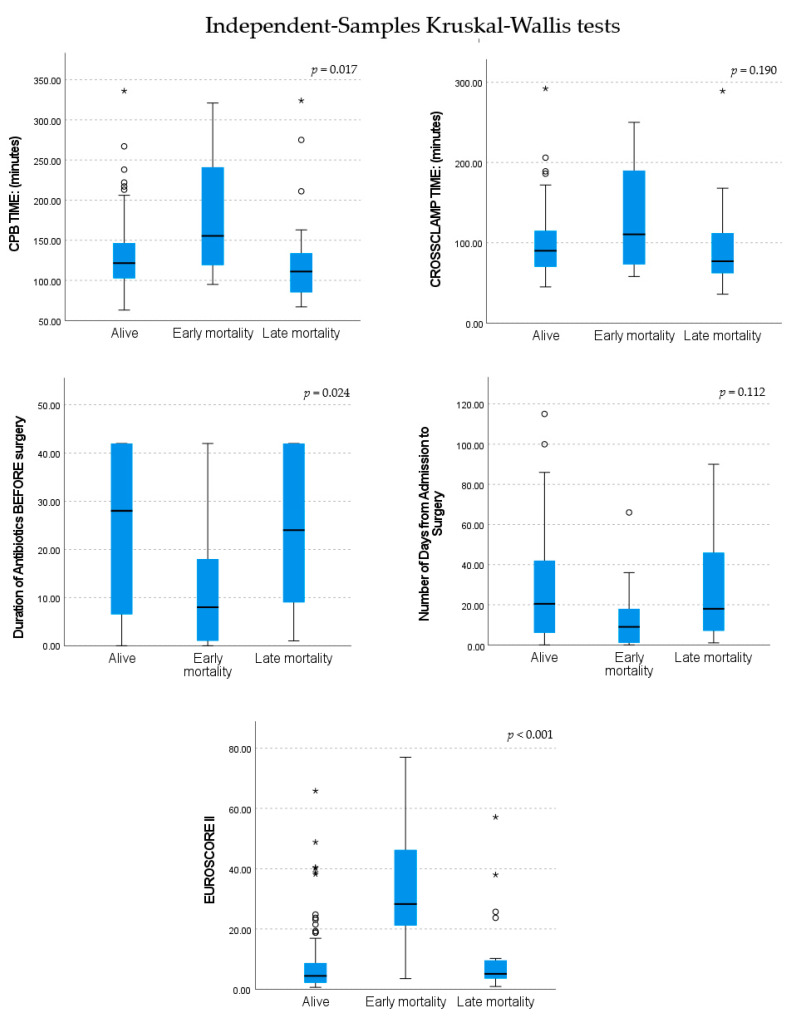
Various factors affecting mortality. The (o) marks individual data points that are considered outliers based on the statistical criteria (more than 1.5 times the interquartile range (IQR), above the third quartile (Q3) or below the first quartile (Q1)). The (*) symbol indicates extreme outliers and is defined as values that lie beyond 3 times the interquartile range (IQR) from the upper or lower quartiles.

The most notable early in-hospital complications associated with mortality were pneumonia (*p* = 0.012) and prolonged intubation (*p* < 0.001). In the late post-operative period, patients that presented with recurrence of IE had a significantly worse outcome (*p* = 0.002). A more detailed breakdown of complications and analysis of early and late mortality is shown in Table 5 and Figure 4. The effect of different organisms and the operative technique used (valve replacement or valve repair) on mortality, are tabulated in Table 6.

**Figure 5:**
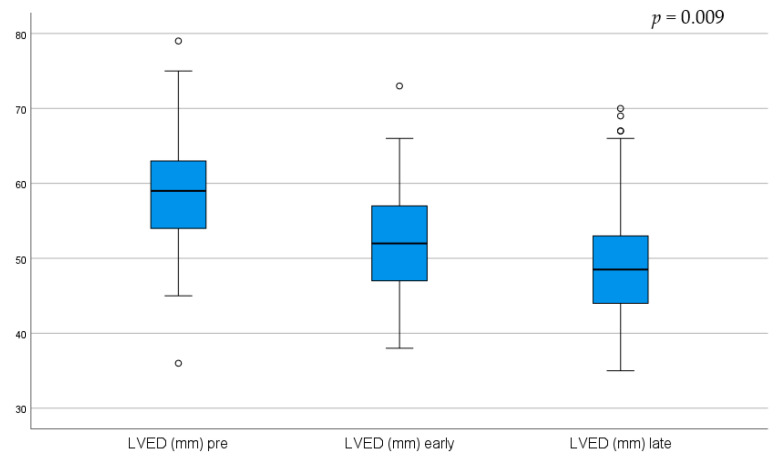
Changes in echo measured Left Ventricular End-diastolic Volume (LVEd) at 3 time points.

**Figure 6:**
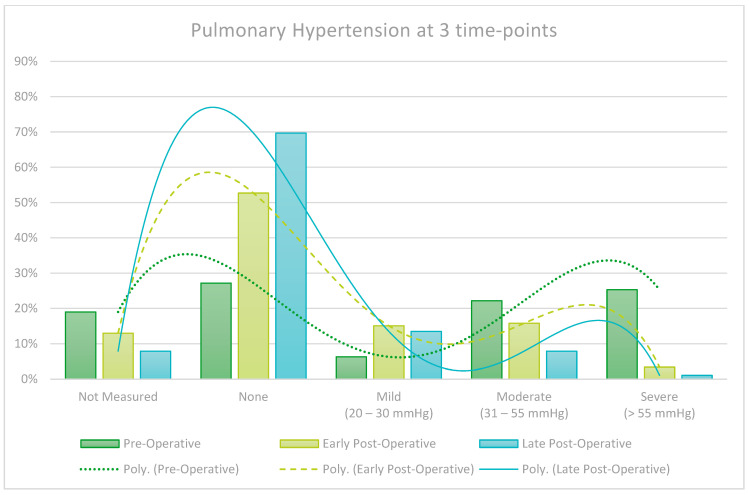
Changes in echo measured Pulmonary Hypertension at 3 time points.

**Figure 7:**
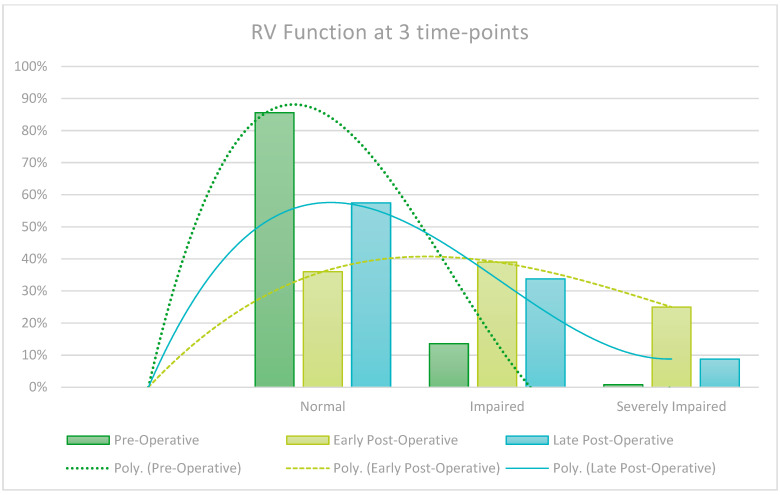
Right Ventricular (RV) Function at 3 time points.

**Figure 8:**
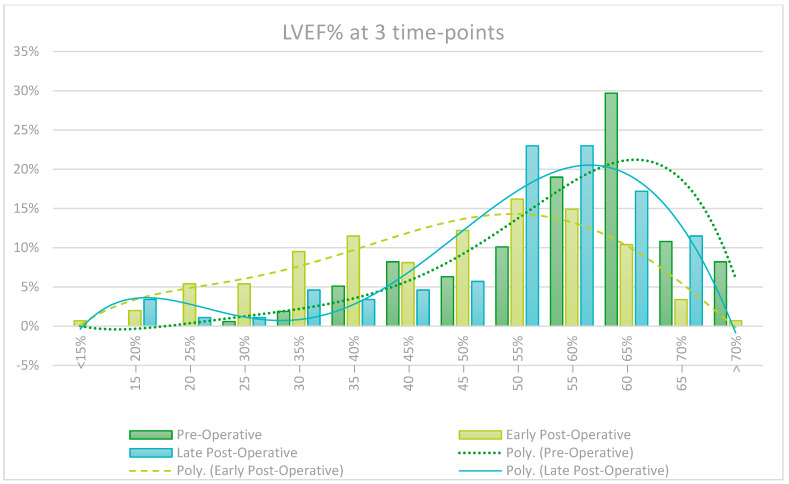
Left Ventricular Ejection Fraction (LVEF%) at 3 time points.

Routine late follow-up echoes are not carried out on all patients and are guided by clinical judgement, creating a risk of selection bias. It is however interesting to note that patients show a notable decrease in early post-operative Left and Right Ventricular Function (LVEF/RVEF), but patients that did in fact get a late echo recovered almost fully by the late post-operative assessment. Left Ventricular End Diastolic Volume (LVEd) also significantly decreased from pre-operatively and continued to do so further in the late post-operative period. These findings are graphically represented in the Figure 5, Figure 6, Figure 7 and Figure 8.

## 4. Discussion

In this cohort of 160 patients, the most significant factors leading to increased mortality were the urgency of intervention, critical pre-operative state, and the presence of single- and multi-organ failure. Other factors included older age, higher pre-operative EuroScore assessment, ongoing pre-operative sepsis, paravalvular extension of the infection, pneumonia, prolonged ventilation, recurrence of infective endocarditis, and the need for repeat valve surgery in the late post-operative period. These findings emphasize the complexity of the clinical management of often seriously ill patients and the multitude of clinical factors that need to be considered in the medical and surgical management of patients with IE requiring surgical intervention. A high number of patients required “Urgent” surgery (56%), but the median time before surgery is 18.5 days. Further discussion and explanation of these findings are noted in the Supplementary Materials.

The outcomes observed in this cohort, as a representation of a low-to-middle-income country (LMIC), compare very well to data from international studies representing high-income countries. An article published in 2013 by Chirillo et al., from Treviso, Italy, reported an in-hospital mortality of 13% and a 3-year mortality of 16% in a cohort of 190 patients undergoing left-sided heart valve surgery for IE [14]. This compares very well with the 8.8% early (<30 days) mortality and 13.1% late (>30 days) mortality observed in this cohort.

In the cohort, with confirmed or suspected infective endocarditis, most patients were previously healthy individuals with a low prevalence of co-morbidities. HIV was present in 15.7% of patients, which is in contrast to data from a recent similar study (2021) by Gwila et al. (2021) at the University of the Free-State, which reported an HIV-positive incidence of 31% in patients with IE requiring cardiac valve surgery [27]. The prevalence observed in our cohort, however, correlates well with data from the Western-Cape Government statistics (2022), stating the incidence of HIV in the local population at 7.3% and 13.4% for males and females, respectively [28]. No significant difference in surgical outcomes in these patients was observed. Older patients and those with higher urgency of intervention had an increased risk for both early and late mortality. Gender did not show a significant difference in outcome. The salvage group had a 100% mortality rate, prompting a debate regarding ethical and resource considerations for the operative and ICU care of these patients. Bearing in mind that patients with a surgical indication who did not undergo surgery, or were declined for surgery on clinical grounds, also showed a similar dismal outcome in a recent prospective cohort study at Tygerberg Hospital by Pecoraro et al. (2022) [13]. An inherent selection bias should also be considered in this patient subgroup, as salvage patients are usually critically ill and the patients who declined surgery are assessed, on pre-operative risk stratification, to be too ill to survive surgical intervention. New York Heart Association (NYHA) functional status showed a significant relation to early and late mortality, with the NYHA IV group having the highest risk. A critical pre-operative state, even with single organ dysfunction, significantly increased the risk of mortality in both early and late operative periods. Co-morbidities did not significantly affect mortality, except for chronic lung disease, which showed a marginal increase in risk but with small numbers, making the clinical significance debatable.

This study found no statistically significant difference in outcomes between mitral and aortic valve intervention, or if concomitant procedures were performed. This finding is interesting and warrants further investigation as previous research by Østergaard et al. (2020) found a higher mortality risk with mitral valve surgery, in comparison to aortic valve surgery in patients with IE [29]. The small sample size may have affected the results, and ongoing sepsis contributes greatly to mortality regardless of the affected valve.

EuroScore II was highly associated with early post-operative mortality but not with long-term mortality. The median EuroScore II of 4.8% was significantly lower than the early mortality (<30 days) of 8.8% that was observed. This is in keeping with findings from several other studies, including articles by Patrat-Delon et al. (2016) and Koshy et al. (2018), which found that EuroScore II calculations underestimate post-cardiac surgery mortality in certain patient subgroups of patients with IE [30,31].

Blood culture-negative endocarditis (BCNIE) was still a frequent observation in this cohort of patients. Since the data collection period has ended, a new article was published by Pecoraro et al. (2021) highlighting the prevalence of Bartonella species and Mycoplasma species as the most common organisms as causes for previously documented BCNIE within the Tygerberg hospital setting. Previously routine blood culture investigations did not specifically investigate these organisms [8]. A new protocol was adopted in November 2019, whereby all patients with preliminary BCNIE undergo further PCR testing for Bartonella, Mycoplasma, Legionella, Brucella, and Coxiella species to help in the identification of a causative organism for IE. Serological samples are collected pre-operatively and valve tissue samples are sent at the time of surgery for a full PCR array assessment for all patients. This has significantly reduced the incidence of BCNIE in Tygerberg Hospital and has since shown great benefit in aiding goal-directed antibiotic management in surgical IE patients. The incidence of BCNIE in a prospective cohort was shown to be significantly lower compared to a retrospective cohort since the adaptation of the protocol change in Tygerberg Hospital (62.7% vs. 42.1% (*p* = 0.039)). In up to 86.2% of patients, a cause could be established in the prospective group, using non-culture techniques. This benefit is substantiated by the observation of a downward trend in the in-hospital mortality of the prospective cohort (14.2%) compared to a retrospective cohort in-hospital mortality (23.4%) (*p* = 0.35) [32].

This study found no statistically significant association between aortic cross-clamp time and post-operative mortality, but there was a noticeable trend towards longer cross-clamp time in the early mortality group. Longer cross-clamp time is typically associated with more complex surgical repairs. A longer cardiopulmonary bypass time was significantly associated with increased mortality risk and is often also associated with worse coagulopathic tendencies, cardiac stunning, systemic inflammatory response, and acute kidney injury, which may also independently contribute to worse outcomes [33,34]. Indications for surgery as a risk for mortality were difficult to interpret, as patients often had multiple indications, but ongoing sepsis and paravalvular extension of infection to the aortic root or para-aortic region were associated with increased mortality.

Pre-operative laboratory parameters, including White Cell Count (WCC), Haemoglobin (HB), and C-Reactive Protein (CRP), were assessed and higher CRP values were observed in patients at higher risk for early mortality, potentially indicating ongoing sepsis. No specific association between organism prevalence and mortality outcomes was observed in this cohort.

Early in-hospital complications are common after major cardiac surgery but were observed to be generally low in the Tygerberg Cardiothoracic unit and may be indicative of good post-operative care. Pneumonia and prolonged ventilation were independent risk factors for increased mortality, while other parameters investigated did not show statistical significance. Late complications showed that IE relapse and the need for recurrent valve surgery were statistically significant risk factors for increased mortality, highlighting the importance of prophylaxis and prevention of re-infections [24,25].

Kaplan–Meier analysis observed a mean overall survival probability period of 100.1 ± 4.7 months (8.3 years). Kaplan–Meier analysis further indicated that the urgency of intervention was highly significant, with a higher risk of early mortality in the salvage group. The mean survival of the other three groups declined progressively, correlating with the higher risk expected with more urgent surgery. This is consistent with a previous study by Rankin et al. that also showed a higher odds ratio (2.11) for mortality in patients with acute IE compared to elective IE valve surgery [35].

In this study, survival rates were significantly affected by clinical functional assessment, especially in patients with NYHA IV functional class. Patients in this group were critically ill and had high mortality rates compared to other groups, highlighting the importance of optimizing patients’ medical status prior to surgery. In a comparable study conducted by Pizzino et al. (2024) in Italy, infective endocarditis (IE)-related heart failure and embolic events were found to be independently associated with an increased risk of major adverse events. However, their cohort exhibited notable differences compared to our study. Specifically, 50% of their patients had prosthetic valve endocarditis, whereas only 8.75% (n = 14) of our patients had a prosthetic valve in situ at the time of presentation. Additionally, the incidence of embolic events in our cohort was lower at 23.75% (n = 38), compared to the 38.2% observed in their study. These differences highlight the variability in patient populations and underscore the need for individualized patient care and protocol development tailored to the specific challenges of local populations [36].

Mitral and aortic repair techniques were found to have similar outcomes, with a trend towards better survival with mitral valve repair. A systematic review by Feringa et al. (2007) underlined that mitral valve repair was possible in patients presenting with mitral valve endocarditis, with repair being associated with lower in-hospital and long-term mortality [16]. Since this review, those findings have been supported by various published results: Rostagno et al. from Italy (2017) and El-Gabry et al. from Germany (2019) [15,37,38].

LVEF decreased in the early post-operative period but recovered to near pre-operative values on late post-operative assessment, an effect likely due to cardiac stunning associated with cardiopulmonary bypass in the early post-operative period. This study found that Left Ventricular End-diastolic (LVEd) measurement showed progressive improvement from the pre- to early post- and late post-operative period, indicating cardiac remodeling and improved volume load on the myocardium during diastole. Pulmonary hypertension also showed a trend of improvement over time. Right Ventricular (RV) failure was observed in the early post-operative period but recovered remarkably over time, also likely due to the effect of peri-operative cardiac stunning and subsequent remodeling during the recovery period.

### Limitations and Challenges

A significant limitation of this study is the inherent bias related to its retrospective and observational nature. Exceptional completeness of data could be achieved, but occasional missing information or absence of clinical recordkeeping impaired data collection and integrity. Despite using a multidisciplinary team approach, the indications and timing of surgery are based on clinical judgment and add to the heterogenicity of patient management. A further major limitation is that the information in relation to IE patients with surgical indications, who were not deemed to be operative candidates and thus declined surgical intervention, is not known or well documented, and makes assessment of this specific patient subgroup impossible.

## 5. Conclusions

In South Africa, infective endocarditis is still a common indication for heart valve surgery despite medical advancements. This study aimed to identify factors affecting patient outcomes at TBH hospitals. The limited sample showed good overall outcomes for both valve repair and replacement. Antibiotic therapy and pre-operative optimization were crucial for better surgical outcomes. Early mortality correlated with EuroScore II pre-operative mortality risk assessment, and several factors associated with increased early mortality were identified. This study provided insight into long-term expected survival in the Western Cape.

This study found that salvage procedures or critical illness with multi-organ failure prior to surgery were associated with poorer outcomes and required ethical considerations. There is a high rate of culture-negative IE, which makes antibiotic stewardship difficult, but newer protocols and additional testing will likely yield improved goal-directed management in surgical IE patients.

The operative outcome of infective endocarditis at Tygerberg Hospital compares well with international standards, despite unique challenges faced by South Africa.

## Figures and Tables

**Table 1:** Basic findings and relevant parameters of cohort (n = 160).

General Parameters		Percentage (Number/Range)
	Confirmed acute infective endocarditis	75.0% (120)
	Suspected acute infective endocarditis	12.5% (20)
	Sub-acute (fully treated, elective surgery)	12.5% (20)
	Number of primary surgeons	11
	Intra-aortic balloon pump required	3.8% (6)
	Admission to surgery	18.5 days (0–115)
	Admission to discharge	54.0 days (7–157)
	Surgery to discharge	29.0 days (0–98)
	Hours intubated post-operatively	18 h (6–1464)
	Blood loss within first 24 h	350 mL (50–2250)
**Follow-up**		
	Post-operative follow-up period	41.0 months (1–131)
	Recurrence of infective endocarditis during follow-up	6.9% (11)
	Repeat valve surgery required during follow-up	8.1% (13)
**Mortality**		
	Alive	72.5% (116)
	Demised—Early (within 30 days)	8.8% (14)
	Demised—Late (after 30 days)	13.1% (21)
	Lost to follow-up, but still alive according to home affairs	5.0% (8)
	Lost to follow-up, unknown if alive	0.6% (1)
**Cultures**		
	Pre-op blood culture positive for organism	47.5% (76)
	Valve culture positive for organism	21.3% (34)
**Histology**		
	Acute infection	39.4% (63)
	Chronic inflammation	30.0% (48)
	Myxoid degeneration	24.4% (39)
	No active or chronic infection/inflammation	38.8% (62)
	Other findings	5.6% (9)
**Echo features pre-operatively**		
	Rheumatic valves	41.8% (66)
	Vegetations visualized	75.9% (120)
	Paravalvular extension	15.9% (25)

**Table 2:** Demographics and the effect on mortality.

				Alive (n = 116)	<30-Day Mortality (n = 14)	>30-Day Mortality (n = 21)	*p*-Value
**Demographics**							
	Mean age (years)			36.2	44.1	40.3	0.04
	Median EUROSCORE2			4.4% (116)	28.3% (14)	5.1% (21)	<0.001
	BMI—Body Mass Index (kg/m^2^)			23.4879	23.6857	23.9571	0.090
	BSA—Body surface area (m^2^)			1.7560	1.7143	1.7333	0.735
**Gender:**							**0.340**
	Male			80.0% (84)	8.6% (9)	11.4% (12)	
	Female			69.6% (32)	10.9% (5)	19.6% (9)	
**Type of valve**							**0.625**
	Native			76.6% (105)	8.8% (12)	14.6% (20)	
	Prosthetic valve in situ			78.6% (11)	14.3% (2)	7.1% (1)	
**Functional status**							**<0.001**
	NYHA * I			83.3% (5)	0	16.7% (1)	
	NYHA II			91.8% (45)	0	8.2% (4)	
	NYHA III			82.7% (43)	7.7% (4)	9.6% (5)	
	NYHA IV			53.5% (23)	23.3% (10)	23.3% (10)	
**Comorbidities**							
	Hypertension		n = 41	78.0% (32)	7.3% (3)	14.6% (6)	0.901
	Diabetes Mellitus		n = 7				0.791
		Insulin		75% (3)	0	25% (1)	
		Oral only		100% (3)	0	0	
	Smoking		n = 70				0.376
		Current smoker		73.8% (45)	14.8% (9)	11.5% (7)	
		Ex-smoker (>6 months)		88.9% (8)	0	11.1% (1)	
	Alcohol abuse		n = 19	57.9% (11)	21.1% (4)	21.1% (4)	0.061
	Illicit substance abuse		n = 20	85.0% (17)	0	15.0% (3)	0.332
	HIV ** Positive		n = 23	65.2% (15)	13.0% (3)	21.7% (5)	0.194
	Chronic lung disease		n = 25	60.0% (15)	20.0% (5)	20.0% (5)	0.051
	Poor mobility		n = 6	83.3% (5)	16.7% (1)	0	0.632

* NYHA: New York Heart Association Classification; ** HIV: Human Immune Deficiency Virus.

**Table 3:** Pre-operative factors influencing mortality.

				Alive (n = 116)	<30-Day Mortality (n = 14)	>30-Day Mortality (n = 21)	*p*-Value
**Time-related factors**							
	Days from admission to surgery			20.50 [6.0–42.0]	9.00 [1.0–18.0]	18.00 [7.0–46.0]	0.112
	Days antibiotics before surgery			28.00 [6.5–42.0]	8.00 [1.0–18.0]	24.00 [9.0–42.0]	0.024
	Aortic cross-clamp time in minutes			90.00 [70.0–115.0]	110.50 [73.0–190.0]	77.00 [62.0–112.0]	0.190
	Cardiopulmonary bypass (CPB) time in minutes			121.50 [102.5–146.5]	155.50 [119.0–241.0]	111.00 [85.0–134.0]	0.017
**Urgency of intervention**			**n = 151**				**<0.001**
	Stable			88.9% (16)	-	11.1% (2)	
	Urgent			84.7% (72)	3.5% (3)	11.8% (10)	
	Emergency			66.7% (28)	14.3% (6)	19.0% (8)	
	Salvage			-	83.3% (5)	16.7% (1)	
**Cardiac function**			**n = 149**				**0.549**
	LVEF * < 50%			76.5% (26)	5.9% (2)	17.6% (6)	
	LVEF * ≥ 50%			77.4% (89)	10.4% (12)	12.2% (14)	
**Organ dysfunction**			**n = 151**				
	Peri-operative cardiac failure			70.6% (72)	12.7% (13)	16.7% (17)	0.025
	Inotropes required pre-induction			61.5% (24)	17.9% (7)	20.5% (8)	0.023
	Intubated/CPAP ** required pre-induction			55.6% (15)	37.0% (10)	7.4% (2)	< 0.001
	Acute renal failure during admission			70.3% (45)	17.2% (11)	12.5% (8)	0.016
	Chronic renal impairment/residual with discharge			43.5% (10)	39.1% (9)	17.4% (4)	0.001
**Critical pre-operative state**			**n = 150**				**< 0.001**
	No			81.3% (100)	4.1% (5)	14.6% (18)	
	Yes			55.6% (15)	33.3% (9)	11.1% (3)	
**Laboratory parameters and mortality**							
	White cell count (WCC) (X109/L)		n = 150	7.61 [5.95–9.69]	9.87 [7.80–12.0]	8.46 [6.50–9.80]	0.078
	C-reactive protein (CRP) (mg/L)		n = 116	43.0 [17.0–88.0]	99.5 [70.0–197.5]	39.0 [12.3–118.0]	0.064
	Haemoglobin (HB) (g/dL)		n = 151	11.15 [9.65–12.5]	10.80 [8.8–11.8]	10.90 [9.8–11.6]	0.680

* LVEF: Left Ventricular Ejection Fraction; ** CPAP: Continuous Positive Airway Pressure.

**Table 4:** The effect of surgical factors on mortality.

			Alive (n = 116)	<30-Day Mortality (n = 14)	>30-Day Mortality (n = 21)	*p*-Value
**Indication for surgery**		**n = 151**				
	Acute severe MR/AR *, with signs of CCF **		75.4% (89)	9.3% (11)	15.3% (18)	0.659
	Large vegetation (>10 mm)		76.2% (32)	11.9% (5)	11.9% (5)	0.737
	Embolic event during first 2 weeks of AB *** therapy		81.6% (31)	7.9% (3)	10.5% (4)	0.761
	Ongoing sepsis, despite adequate AB therapy		61.1% (11)	27.8% (5)	11.1% (2)	0.022
	Sub-acute Infective Endocarditis with CCF unresponsive to medical therapy		86.7% (13)	0	13.3% (2)	0.506
	Paravalvular extension of infection (abscess)		62.5% (20)	18.8% (6)	18.8% (6)	0.046
**Type of intervention**						**0.929**
	First time valve surgery	n = 135	76.3% (103)	8.9% (12)	14.8% (20)	
	First redo	n = 13	76.9% (10)	15.4% (2)	7.7% (1)	
	Second redo	n = 2	100% (2)	-	-	
	Third + fourth redo	n = 1	100% (1)	-	-	
**Primary valve intervention**						**0.409**
	Mitral valve	n = 74	82.9% (58)	5.7% (4)	11.4% (8)	
	Aortic valve	n = 60	71.9% (41)	10.5% (6)	17.5% (10)	
	Both mitral AND aortic valves	n = 26	70.8% (17)	16.7% (4)	12.5% (3)	
**Concomitant procedures**						
	CABG ****		75.0% (3)	25.0% (1)	-	0.434
	Tricuspid valve annuloplasty/replacement		33.3% (1)	33.3% (1)	33.3% (1)	0.174
	Vein patch leaflet repair		100% (7)	-	-	0.330
	Other procedures		65.0% (13)	15.0% (3)	20.0% (4)	0.396

* MR/AR: Mitral Regurgitation/Aortic Regurgitation; ** CCF: Congestive Cardiac Failure; *** AB: Antibiotic; **** CABG: Coronary Artery Bypass Grafting.

**Table 5:** Post-operative factors influencing mortality outcomes.

				Alive (n = 116)	<30-Day Mortality (n = 14)	>30-Day Mortality (n = 21)	*p*-Value
**Early in-hospital post-operative complications**							
	Excessive bleeding (>500 mL in 24 h)		n = 48	68.8% (33)	14.6% (7)	16.7% (8)	0.197
	Re-operated for bleeding		n = 13	61.5% (8)	15.4% (2)	23.1% (3)	0.531
	Re-operated for pericardial effusion		n = 7	100% (7)	0	0	0.234
	Pneumonia		n = 24	54.2% (13)	16.7% (4)	29.2% (7)	0.012
	Wound sepsis		n = 12	83.3% (10)	0	16.7% (2)	0.606
	Prolonged intubation (>24 h)		n = 26	50.0% (13)	30.8% (8)	19.2% (5)	<0.001
	Neurological Event (CVA/TIA) *						0.771
		(Pre-Operative)	n = 36	83.3% (30)	5.6% (2)	11.1% (4)	
		(Post/Peri-Operative)	n = 13	76.9% (10)	15.4% (2)	7.7% (1)	
	Peripheral Emboli						0.795
		(Pre-Operative)	n = 10	80.0% (8)	10.0% (1)	10.0% (1)	
		(Post/Peri-Operative)	n = 2	100.0% (2)	0	0	
	Atrial Fibrillation						0.338
		(Pre-Operative/Chronic)	n = 9	100.0% (9)	0	0	
		(Post/Peri-Operative)	n = 12	58.3% (7)	33.3% (4)	8.3% (1)	
	Permanent Pacemaker						0.121
		(Pre-Operative/Chronic)	n = 1	0	0	100% (1)	
		(Post/Peri-Operative)	n = 4	75.0% (3)	25.0% (1)	0	
**Late post-surgery discharge complications**							
	Re-admitted with surgery related complication		n = 15	66.7% (10)	0	33.3% (5)	0.123
	Re-infection diagnosed as infective endocarditis		n = 11	45.5% (5)	0	54.5% (6)	0.002
	Repeat valve surgery required		n = 13	61.5% (8)	0	38.5% (5)	0.038
	Re-operation for paravalvular leak		n = 1	100% (1)	0	0	1.0

* CVA/TIA: Cerebral Vascular Accident/Transient Ischemic Attack.

**Table 6:** Other factors influencing mortality, irrespective of time-period.

				Alive	Dead	*p*-Value
**Organism**						
	Streptococci Viridans		n = 14	100% (14)	0% (0)	0.041
	Group D Streptococci		n = 7	57.1% (4)	42.9% (3)	0.349
	Streptococcus other		n = 12	83.3% (10)	16.7% (2)	0.740
	Staphylococcus		n = 33	81.8% (27)	18.2% (6)	0.644
	Hacek organisms		n = 6	50% (3)	50% (3)	0.119
	Fungi		n = 3	66.7% (2)	33.3% (1)	1.000
	Other gram positives		n = 11	63.6% (7)	36.4% (4)	0.258
	Other gram negatives		n = 23	82.6% (19)	17.4% (4)	0.608
	All cultures negative		n = 67	76.1% (51)	23.9% (16)	0.699
**Repair vs. replacement**						
	MITRAL VALVE					0.076
		Repair	n = 33	90.9% (30)	9.1% (3)	
		Replacement	n = 67	76.1% (51)	23.9% (16)	
	AORTIC VALVE					0.793
		Repair	n = 3	66.6% (2)	33.3% (1)	
		Replacement	n = 83	73.5% (61)	26.5% (22)	

## Data Availability

Data cannot be made publicly available as it is prohibited and regulated by the POPI (Protection of Personal Information Act 4 of 2013) in South Africa. A written request for specific information may be sent to the corresponding author, who will evaluate and provide further information as possible.

## Supplementary Materials

The following supporting information can be downloaded at https://www.mdpi.com/article/10.3390/jcm13175226/s1, “Appendix to the manuscript: The Surgical Outcome of Infective Endocarditis in South Africa over 10 years: a Retrospective Review”. References [39,40,41,42,43,44,45,46,47] are cited in the Supplementary Materials.
