# Supplementary material for: The Surgical Outcome of Infective Endocarditis in South Africa over 10 Years: A Retrospective Review
Source: J Clin Med. 2024 Sep 3;13(17):5226. doi: 10.3390/jcm13175226 (PMC11395924; doi:10.3390/jcm13175226)
Supplement: Supplementary file 1 [file jcm-13-05226-s001.zip › jcm-3132969-supplementary.pdf]

APPENDIX TO THE MANUSCRIPT:

The Surgical Outcome of Infective Endocarditis in South Africa over 10 years: a Retrospective Review

In addition to obtaining patient information form Tygerberg Hospital's Electronic Content Management (ECM) system and physical hospital files, the study team attempted to contact the outstanding patients on all available telephone numbers on record to confirm their alive status. The patients for whom no clinical data could be traced or verified were coded as lost-to-follow-up (n=9). The patients that were deemed lost to follow-up, were excluded for the analysis looking at the parameters affecting early and late mortality as their follow-up information was incomplete. The South African National Department of Home Affairs (DOHA) was consulted to verify the alive status of these 9 patients, of whom 8 could be confirmed to still be alive. However, for the Kaplan Meier mortality over time assessment, they were included in the analysis and captured as still being alive. These 8 patients were also included in the pre- and peri-operative data analysis regarding their hospital admission and surgery, as this data could be obtained from the operative notes and hospital administration system. If a mortality or early complication occurred during the admission period, it was documented and included in the analysis. The data were censored from the time of last healthcare contact, and late complications that might have occurred after this censor date, were thus also excluded from analysis.

Unique study identifiers were assigned to the initial list of possible participants for anonymous data collection, which could be cross-referenced through a password-protected MS Excel file. After applying exclusion and inclusion criteria, a new REDcap record number was assigned to the final list of 160 participants. A 172-parameter database was created using REDcap, divided into 10 sections including demographics, pre-operative history, intervention, surgery details, complications, and three sections for echocardiographic data. A paper-based template was used to capture data initially, which was later entered into the REDcap electronic database for easy data extraction and statistical analysis. Despite being challenging, the study achieved excellent completeness through various sources and systems.

At Tygerberg hospital, registrar training is a routine part of daily activities. A senior consultant performs valve repair surgeries, while replacement surgeries are performed by registrars under consultant supervision or by consultants themselves. Surgeon experience is not expected to impact the data. Post-operative care is provided in a 14-bed Cardiothoracic ICU by consultants and registrars. Mild hypothermia is used during surgery as per the standard protocol, with a core temperature of 32°C during bypass and rewarming to 36°C prior to decannulation from cardiopulmonary bypass (CPB). An underbody warming air blanket is used until a consistent body temperature is maintained in the unit.

In this study, the transfusion trigger for donor packed red blood cells (RBC) are typically set at a Haemoglobin (HB) value of 8.0g/dL or a haematocrit of less than 29% after the patient has been weaned from CPB and the auto transfusion of Cellsaver® blood has been completed. Excessive bleeding over 500ml within the first 24 hours after surgery warrants medical correction of coagulation with blood products or surgical re-exploration. Acute kidney injury is defined according to KDIGO (Kidney Disease Improving Global Outcomes) guidelines and classified by serum creatinine levels, urine volume, and severity.[39] Chronic kidney disease is defined as kidney damage or eGFR less than 60 ml/min/1.73m^2 for 3 months or more, and further classified by glomerular filtration and albuminuria levels.[40,41] A standard serum creatinine cut-off of 150 umol/L was used for all patients, and discharge with residual serum creatinine above this value was considered a degree of chronic renal impairment.

Critical pre-operative state and timing of surgery classification were determined by using the EUROSCORE II scoring system definitions. Patients who require surgery on the current admission for medical reasons and cannot be discharged without a definitive procedure is regarded as an urgent procedure. An operation is deemed emergency if it is done before the beginning of the next working day after the decision to operate was made. A salvage procedure is defined as a patient requiring cardiopulmonary resuscitation (external cardiac massage) enroute to the operating theatre or prior to the induction of anaesthesia.[42]

In Tygerberg Hospital the protocol regarding timing of surgery is based on a holistic assessment of the patients' clinical condition, by the combined heart team.[13] If the patient is clinically stable, the aim is to complete the full 42days of IV antibiotics, prior to doing the valve surgery. The cardiac tissue is often very friable and inflamed during the acute phase of IE, requiring careful suture placement. Longer antibiotic therapy aid in improving tissue quality, by reducing the bacterial load and inflammation. Clinical deterioration prior to the completion of the 42days antibiotics, peri-valvular extension or persistently large high embolic risk vegetations, often require urgent surgical intervention prior to the completion of this period and are guided by ESC and EACTS guidelines [24]. In these cases, the patient will have to complete the remainder of the 42days of IV antibiotics post-surgery and explains the longer than expected post-surgery to discharge hospital admission stay (29.0 days) duration.

Post-operative antibiotic therapy is further guided by intra-operative tissue assessment and evidence of ongoing active infection. If significant residual vegetations is visualized, source control is deemed to only be established at the time of surgery, and a full 42day course will be restarted. Active infection on histology or a positive valve tissue culture, or an organism not susceptible to the pre-operative blood culture identified sensitivities, will also prompt the re-initiation of a full 42 days of postoperative organism sensitivity guided antibiotic therapy. If a susceptible organism, with definitive sensitivities is confirmed on microbiological culture or PCR investigation, the antibiotic regime would be de-escalated according to the recommendation from the infectious disease and microbiology consultant on the IE team.

Despite advances in modern medicine, and better laboratory testing the prevalence of culture negative infective endocarditis is still very high within the WC setting. In some countries, the identification of causative organisms is as high as 90%, using various advanced PCR assays and laboratory testing [43]. Within the Tygerberg hospital setting, resource limitations preclude the performance of all the expensive investigations routinely, and they are limited to funded research or complex cases. Cultures and antimicrobial susceptibility testing will guide the choice of antibiotics once they become available. The standard protocol dictates that 3x blood cultures, using the Standard BacT/ALERT® FA Plus (Fastidious Antimicrobial Neutralization Plus Media) blood-culture bottle, should be taken at least 30 min apart. This should be done, from 3 different sites using a sterile technique, and completed prior to initiation of antibiotic therapy. Due to Tygerberg hospital being a tertiary level care referral centre, these cultures are often performed at primary or secondary care hospitals, prior to the transfer of the patient. The accuracy and quality of these samples can thus often not be verified accurately, and often do not meet the criteria required. Due to initiatives world-wide that advocate early antibiotic therapy to decrease mortality in septic patients, as seen in the "Surviving Sepsis campaigns", junior doctors would often start treating sepsis empirically, prior to sufficiently completing the necessary blood cultures [44]. Unfortunately, this results in a large number of patients with confirmed IE, but without a specific organism identified pre-operatively, representing the large incidence of BCNIE.

Empirical therapy will usually be similar to what is given to culture-negative cases. For native valve endocarditis intravenous Penicillin G (5-6 million Units q6hrly IV for 6 weeks), Cephazolin (2g q6hrly IV for 6 weeks) and Gentamicin (1 mg/kg q8h IV for 2 weeks). If a Staphylococcus is confirmed, the Cephazolin will be replaced with Cloxacillin (2 g q6hg IV for 6 weeks). The antibiotic strategy is guided by the European Society of Cardiology (ESC) and European Association of Cardiothoracic Surgeons (EACTS) guidelines, that are reviewed periodically.[6]

Prosthetic valve endocarditis is treated with a combination of intravenous Vancomycin and oral Rifampicin for 6 weeks in combination with 8 hourly intravenous Gentamicin for the first 2 weeks of the therapy [5]. Patients will not receive out-patient parenteral, antibiotic therapy (OPAT), unless they have completed at least 28 days IV antibiotics, and have a proven organism, e.g. Mycoplasma or Coxiella Brunetti, that is sensitive to oral Doxycycline. However, this has only recently become practice within the TBH setting, and most of the patients in the study have still received the full 6 weeks (42 days) of in-patient IV antibiotics, either at TBH or a secondary referral hospital.

Pre-operative laboratory parameters, including White Cell Count (WCC), Haemoglobin (HB), and C-Reactive Protein (CRP), were assessed and higher CRP values were observed in patients at higher risk for early mortality, potentially indicating ongoing sepsis. No specific association between organism prevalence and mortality outcomes was observed in this cohort.

Early mobilization is one of the most important parameters in decreasing morbidity and mortality in the ERAS (enhanced recovery after surgery) guidelines. It is important that each patient has a good functional status assessment prior to surgery. Infective endocarditis patients often present with embolic phenomenon resulting in cerebral vascular accident (CVA) with complications of a hemiplegia, aphasia, or mycotic aneurysms. This greatly impacts the post operative rehabilitation process and adds additional strain on nursing staff, as well as supporting disciplines like physiotherapists. Silent embolic phenomenon, especially to the brain and spleen, have been reported in up to 20-50% of IE patients on CT and MRI, with 15-30% of patients having symptomatic neurological complication [45,46]. Unfortunately due to resource constraints, routine pre-operative radiological investigation on all IE patients is not feasible, but are guided by clinical findings if any neurological compromise is suspected. TTE has been accepted as a valuable adjunct to visualization of vegetations and vital in the assessment of high embolization risk vegetations. It is routinely used as an assessment tool and vegetations with a linear length longer than 10mm has been associated with a higher in-hospital mortality incidence [26].

Patients with confirmed neurological compromise, will all undergo CT Brain assessment, and the further management is based on the ESC/EACTS 2015 guidelines on IE [43]. If a haemorrhagic CVA has been confirmed on imaging, surgery will be delayed for at least 1 month. If haemorrhage has been excluded, the timing of surgery would be individualized according to the patient's extent of neurological compromise, expected recovery and risk for further embolization. A heart team discussion will guide management, with the aim of optimizing rehabilitation pre-operatively, while limiting the risk for further embolization and maximise the benefit from early surgery [13,26]. Clinical features of cardiac failure and ongoing sepsis would also be taken into consideration. If a mycotic aneurysm is identified, the treatment of the extra-cardiac disease will be guided by Neurosurgery or Vascular surgery – depending on the severity and location involved. If a patient is completely stable, and is deemed a low risk for further embolization, a more conservative intense outpatient rehabilitation program, with elective surgery after 6 months, may be considered in select cases.

TTE assessment is routinely used preoperatively and postoperatively to evaluate cardiac and valvular function in patients undergoing cardiac surgery. All patients receive an early post-operative echo, prior to discharge, after the pericardial drains has been removed. This is to ensure good valve function and to exclude the presence of any paravalvular leaks or significant pericardial effusions. Follow-up is arranged with all patients, receiving a cardiothoracic surgery outpatient clinic booking approximately 6-8 weeks after discharge. Once the patient has fully recovered from his surgery, he will be referred back to the division of Cardiology at Tygerberg hospital, for annual long-term follow-up and surveillance. Routine late follow-up echoes are not done and are guided by clinical judgement if indicated. Patients are provided with extensive counselling on Warfarin compliance, wound care, and general health, and are offered an open-door policy for any concerns during the early operative period. INR monitoring is done at the local community healthcare clinic, and medication therapy is optimised based on the type of valve replacement or repair. Patients with valve replacements are usually prescribed angiotensin converting enzyme (ACE) inhibitors, beta blockers, and Warfarin anticoagulation, while those with valve repairs typically only receive single anti-platelet therapy (Aspirin). Patients who develop

complications requiring further surgical intervention, are represented at a weekly multi-disciplinary team meeting for discussion of possible re-intervention.

Surgical treatment options include valve replacement and repair, with repair showing a better early and late survival advantage in mitral valve endocarditis [16]. A trend towards better survival with mitral valve repair was seen (**Table 6)**. A systematic review by Feringa et al. (2007) underlined that mitral valve repair was possible in patients presenting with mitral valve endocarditis, with repair being associated with lower in-hospital and long-term mortality [16]. Mitral valve repairs are often challenging, and various factors has been shown to affect the durability of the repair and subsequent outcomes, including poor ventricular function, the presence of pulmonary hypertension, atrial fibrillation and the pathophysiology of the mitral incompetence [17].

<table>
<tr><th>INDICATIONS FOR SURGERY</th><th>Class</th><th>CONSIDERATION FOR EARLY SURGERY</th></tr>
<tr><td colspan="3">HEART FAILURE</td></tr>
<tr><td>Severe aortic or mitral valve incompetence
Heart failure (HF) caused by severe aortic or mitral regurgitation, intracardiac fistulae or valve obstruction caused by vegetations. In the absence of clinical HF but with echocardiographic signs of elevated left ventricular end-diastolic pressure (e.g. premature closure of the mitral valve), high left atrial pressure or moderate to severe pulmonary hypertension.</td><td>I</td><td>Cardiogenic Shock, pulmonary oedema, refractory cardiac failure not responding to medical therapy or echo signs of poor haemodynamic tolerance.</td></tr>
<tr><td colspan="3">UNCONTROLLED INFECTION</td></tr>
<tr><td>Locally uncontrolled infection
Perivalvular extension of infection, including abscess, false aneurysms, fistula or enlarging vegetations or new onset heart block</td><td>I</td><td rowspan="3">Persistent positive blood-cultures despite appropriate antibiotic therapy (7-10 days [ESC], 5-7days [AATS]) and adequate control of septic embolic foci.</td></tr>
<tr><td>Infection caused by Fungi or multi-resistant organisms</td><td>I</td></tr>
<tr><td>PVE caused by Staphylococci or non-Hacek gram-negative bacteria</td><td>I</td></tr>
<tr><td>PVE with relapse infection after completing a full course of antibiotics and being culture negative</td><td>IIa</td><td></td></tr>
<tr><td colspan="3">PREVENTION OF EMBOLISM</td></tr>
</table>

**Persistent vegetations (>10mm) after one or more embolic episode despite appropriate antibiotic therapy**	**I**	In order to prevent vegetation emboli, consider the presence of previous embolic events, other complications of IE, the size and mobility of the vegetation, the likelihood of conservative surgery and the duration of antibiotic therapy.
**Isolated large vegetation (>15mm)**	**IIb**	

Table 7 - Indications for Surgery for Infective Endocarditis [26, 43, 47]

**HISTOLOGY**	
Acute infection	39.4% (63)
Chronic inflammation	30.0% (48)
Myxoid degeneration	24.4% (39)
No active or chronic infection/inflammation	38.8% (62)
Other findings	5.6% (9)

Table 8 – Histology and Culture Organism Distribution
